# Expression, homology modeling and enzymatic characterization of a new β-mannanase belonging to glycoside hydrolase family 1 from *Enterobacter aerogenes* B19

**DOI:** 10.1186/s12934-020-01399-w

**Published:** 2020-07-14

**Authors:** Siyu Liu, Tangbing Cui, Yan Song

**Affiliations:** School of Biological Science and Bioengineering, South China University of Technology, Guangzhou, 510006 China

**Keywords:** *Enterobacter aerogenes*, β-mannanase, CBM, GH1 family, Enzymatic characterization

## Abstract

**Background:**

β-mannanase can hydrolyze β-1,4 glycosidic bond of mannan by the manner of endoglycosidase to generate mannan-oligosaccharides. Currently, β-mannanase has been widely applied in food, medicine, textile, paper and petroleum exploitation industries. β-mannanase is widespread in various organisms, however, microorganisms are the main source of β-mannanases. Microbial β-mannanases display wider pH range, temperature range and better thermostability, acid and alkali resistance, and substrate specificity than those from animals and plants. Therefore microbial β-mannanases are highly valued by researchers. Recombinant bacteria constructed by gene engineering and modified by protein engineering have been widely applied to produce β-mannanase, which shows more advantages than traditional microbial fermentation in various aspects.

**Results:**

A β-mannanase gene (*Man1E*), which encoded 731 amino acid residues, was cloned from *Enterobacter aerogenes*. Man1E was classified as Glycoside Hydrolase family 1. The bSiteFinder prediction showed that there were eight essential residues in the catalytic center of Man1E as Trp166, Trp168, Asn229, Glu230, Tyr281, Glu309, Trp341 and Lys374. The catalytic module and carbohydrate binding module (CBM) of Man1E were homologously modeled. Superposition analysis and molecular docking revealed the residues located in the catalytic module of Man1E and the CBM of Man1E. The recombinant enzyme was successfully expressed, purified, and detected about 82.5 kDa by SDS-PAGE. The optimal reaction condition was 55 °C and pH 6.5. The enzyme exhibited high stability below 60 °C, and in the range of pH 3.5–8.5. The β-mannanase activity was activated by low concentration of Co^2+^, Mn^2+^, Zn^2+^, Ba^2+^ and Ca^2+^. Man1E showed the highest affinity for Locust bean gum (LBG). The K_m_ and V_max_ values for LBG were 3.09 ± 0.16 mg/mL and 909.10 ± 3.85 μmol/(mL min), respectively.

**Conclusions:**

A new type of β-mannanase with high activity from *E. aerogenes* is heterologously expressed and characterized. The enzyme belongs to an unreported β-mannanase family (CH1 family). It displays good pH and temperature features and excellent catalysis capacity for LBG and KGM. This study lays the foundation for future application and molecular modification to improve its catalytic efficiency and substrate specificity.

## Background

Mannan, a major hemicellulose, is widely found in plant cell walls, some plant seeds ungi and marine bacteria. It is divided into three classes: linear mannan, glucomannan, galactomannan and galactoglucomannan [1]. The main chains of linear mannan and galactomannan consist of β-1,4-d-mannopyranose residues [2]. The main chains of glucomannan and galactoglucomannan are linked by d-mannopyranose and d-glucopyranose through β-1,4-glycosidic bonds [3]. β-Mannanase (endo β-1,4-d-mannanase, EC 3.2.1.78), one of glycoside hydrolases, catalyzes hydrolysis of linear mannan, galactomannan, glucomannan, and galactoglucomannan through randomly breaking β-1,4-mannosidic linkages [4–6], releasing short β-1,4 mannose-oligosaccharides or glucomannose-oligosaccharides [7]. β-1,4-mannanases are widely distributed in bacteria, fungi, archaea, higher plants and mollusks. Based on the homology of amino acid sequences and phylogenesis, β-1,4-d-mannanases are generally grouped in family GH5, GH26, GH113 and GH134 in CAZy database [1, 8]. GH5 family β-1,4-mannanases are widely distributed in archaea, bacteria, fungi, higher plants and animals, such as β-1,4-mannanases from *Glaciozyma antarctica* PI12 [Sepideh Parvizpour, Jafar Razmara, Aizi Nor Mazila Ramli, Rosli Md Illias, Mohd Shahir Shamsir. Structural and functional analysis of a novel psychrophilic β-mannanase from *G. antarctica* PI12. J Comput Aided Mol Des (2014) 28:685–698], *Bacillus pumilus* GBSW19 [Haoyu Zang, Shanshan Xie, HuijunWu, WeiduoWang, Xiankun Shao, Liming Wu, Faheem Uddin Rajer, XuewenGao. A novel thermostable GH5_7 β-mannanase from *B. pumilus* GBSW19 and its application in manno-oligosaccharides (MOS) production. Enzyme and Microbial Technology 78 (2015) 1–9], *Arabidopsis thaliana* [Yang Wang, Francisco Vilaplana, Harry Brumer, Henrik Aspeborg. Enzymatic characterization of a glycoside hydrolase family 5 subfamily 7 (GH5_7) mannanase from *A. thaliana*. Planta (2014) 239:653–665], and *Trichoderma reesei* [E. Sabini, H. Schubert, G. Murshudov, K.S. Wilson, M. Siika-Aho, M. Penttilä, The three-dimensional structure of a *T. reesei* β-mannanase from glycoside hydrolase family 5, Acta Crystallogr. Sect. D Biol. Crystallogr. 56 (2000) 3–13.]. Fungal β-mannanases are usually grouped in family 5. GH26 family β-1,4-mannanases are mainly present in bacteria and a few of fungi. The reported fungal β-1,4-mannanases in GH26 family include Man26A from *Aspergillus niger* CBS 513.88 [9], MtMan26A from *Myceliophthora thermophila* [10] and AnMan26A from *Aspergillus nidulans* [11] and so on. GH113 family β-1,4-mannanases are derived mainly from bacteria and a very small number of viruses, archaea and eukaryotes, such as AaManA from *Alicyclobacillus acidocaldarius* [12]. GH134 family β-mannanases are mainly from fungi and very few bacteria. Such as *Streptomyces* sp. NRRL B-24484 [13], *A. nidulans* A26 [14] and *Aspergillus oryzae* RIB40 [15]. The homology of the amino acid sequence among the members of the GH5, GH26 and GH113 family is very low. But according to the analysis of three-dimensional structure and catalytic mechanism, the β- mannanases from these three families have TIM (β/α)_8_ barrel structure, and follow the same catalytic mechanism (double-substituted reaction mechanism) [16–18]. A carbohydrate binding module (CBM) is usually present in the hemicellulose degrading enzyme structure, which is an independent region that constitutes the enzyme. A CBM can bind multiple catalytic active domains (CD) and promote carbohydrates binding to it. Most β- mannanases from the CBM1, CBM6, CBM10, CBM31 and CBM35 families have CBM structures [19, 20]. Studies have shown that the binding of CBM and CD can improve structural flexibility of enzyme to make substrate and enzyme better chimeric, thereby increasing the concentration of substrate [21]. A lot of the 3D structures of the catalytic active domains (CD) and family 35 carbohydrate binding modules (CBM) of β-1, 4-mannanases have been resolved (https://www.rcsb.org), and a few examples are displayed in Additional file 1.

Microbial β mannanases have been extensively studied for their gene expression, biochemical and structural characteristics, and various applications. Microbial β-mannanases have been reported from *Bacillus subtilis* N16-5 [22], *A. oryzae* [23], *Richteris Trichoderma* [24], *Aspergillus chalcogenides* [25], *Penicillium* [26] and so on. β-mannanases have showed prospective applications, for instance, pulp decolourizing [27], coffee extract viscosity reducing [28], detergent formulating [29], food quality improving [30, 31] and animal feed nutritional value improving [32]. *Enterobacter aerogenes* B19 is separated from the root soil of rotten stumps, and its sequence of 16S ribosomal RNA gene has been submitted to NCBI database (GenBank accession number: KU500561.1). In the present study, we report a new β-mannanase (Man1E) belonging to CH1 family from *E. aerogenes* B19 and so far no literatures report β-mannanase from CH1 family. We have carried out the homologous modeling for the catalytic module and CBM of Man1E and molecular docking studies to elucidate the three dimensional structure, active site apparatus and enzyme–substrate interaction of this enzyme. We also have successfully cloned and expressed *Man1E* gene in *Escherichia coli*. In addition, we determine biochemical characteristics and substrate specificity of the enzyme.

## Results and discussion

### Cloning and sequence analysis of β-mannanase gene

The β-mannanase gene was successfully amplified from *E. aerogenes* B19 by PCR. The ORF of the β-mannanase gene was 2196 bp in length, encoding 731 amino acids. The molecular mass (Mw) and isoelectric point (pI) of the β-mannanase deduced by ProtParam were 81.72 kDa and 5.91, respectively. A signal peptide from residues 1 to 24 in the sequence was found by SignalP prediction. The amino acid sequence of Man1E was analyzed by BLASTp and it showed high sequence homology with *Enterobacteriaceae* bacteria and *Cronobacter* bacteria, 98.77–99.73% identity with a number of β-mannanases from *Enterobacter ludwigii* and *Enterobacter cloacae* strains, and about 90% identity with other bacterial β-mannanases from several *Cronobacter dublinensis* strains and many *Klebsiella aerogenes* strains. But so far, the identification and characterization of these β-mannanases through purification and heterologous expression have not been reported yet. Man1E displayed 90.42%, 99.73% and 90.42% identity with unreported bacterial β-1, 4-mannanases from *K. aerogenes*, *E. ludwigii* and *E. coli*; 36.69%, 32.28% and 28.00% identity with unreported plant β-1, 4-mannanases from *Solanum lycopersicum*, *Oryza sativa Japonica* Group and *A. thaliana*; and 25.56%, 22.54%, 26.67%, 26.67% and 26.37% identity with reported bacterial GH5 family β-1, 4-mannanases from *Cellvibrio mixtus* (PDB code, 1UUQ-A), *Rhizomucor miehei* (PDB code, 4LYP-A), *S. lycopersicum* (PDB code, 1RH9-A), *Podospora Anserina* (PDB code, 3ZIZ-A) and *T. Reesei* (PDB code, 1QNR-A). The amino acid sequences of above β-mannanases were aligned by Clustal Omega (https://www.ebi.ac.uk/Tools/msa/clustalo/). The result was shown in Additional file 2: Figure S2. Among these β-1, 4-mannanases, seven highly conserved glycines, which were located at 38, 52, 170, 171, 258, 311 and 337 positions in Man1E, were found. The role of these conserved glycines in β-mannanases has not been reported. A highly conserved histidine residue (at 279 position in Man1E) was found and binded to substrates through imidazole nitrogen [33–35]. A highly conserved tryptophan, which was seated at 341 position in Man1E, has been demonstrated to participate in the formation of a hydrophobic platform and bind to substrates in the catalytic region of GH5 family β-1, 4-mannanases [33–35]. Two highly conserved Glutamate residues, which were located at 230 and 309 positions in Man1E, were identified as the acid–base catalytic residue and the nucleophilic catalytic residue in all GH5 family and GH26 family β-1, 4-mannanases [33–35]. An asparagine residue next to the acid–base catalytic residue existed in β-1, 4-mannanases and binded to substrate. Other highly conserved residues included two arginines, two asparticacids, a tyrosine, a histidine, a phenylalanine, a isoleucine and a lysine, which were located at 104, 207,145, 274, 216, 222, 225, 266 and 303 positions in Man1E, respectively. Two motifs, including an “EF(Y)G” motif and an “IM(F/L)A(S)WE(Q)” motif, were found in β-mannanases. The “EF(Y)G” motif was composed of the nucleophilic catalytic residue and next two residues and its effect of on the nucleophilic residue attacking substrates has been still unclear. The IM(F/L)A(S)WE(Q) motif and its function has not been reported in any literatures.

**Fig. 1:**
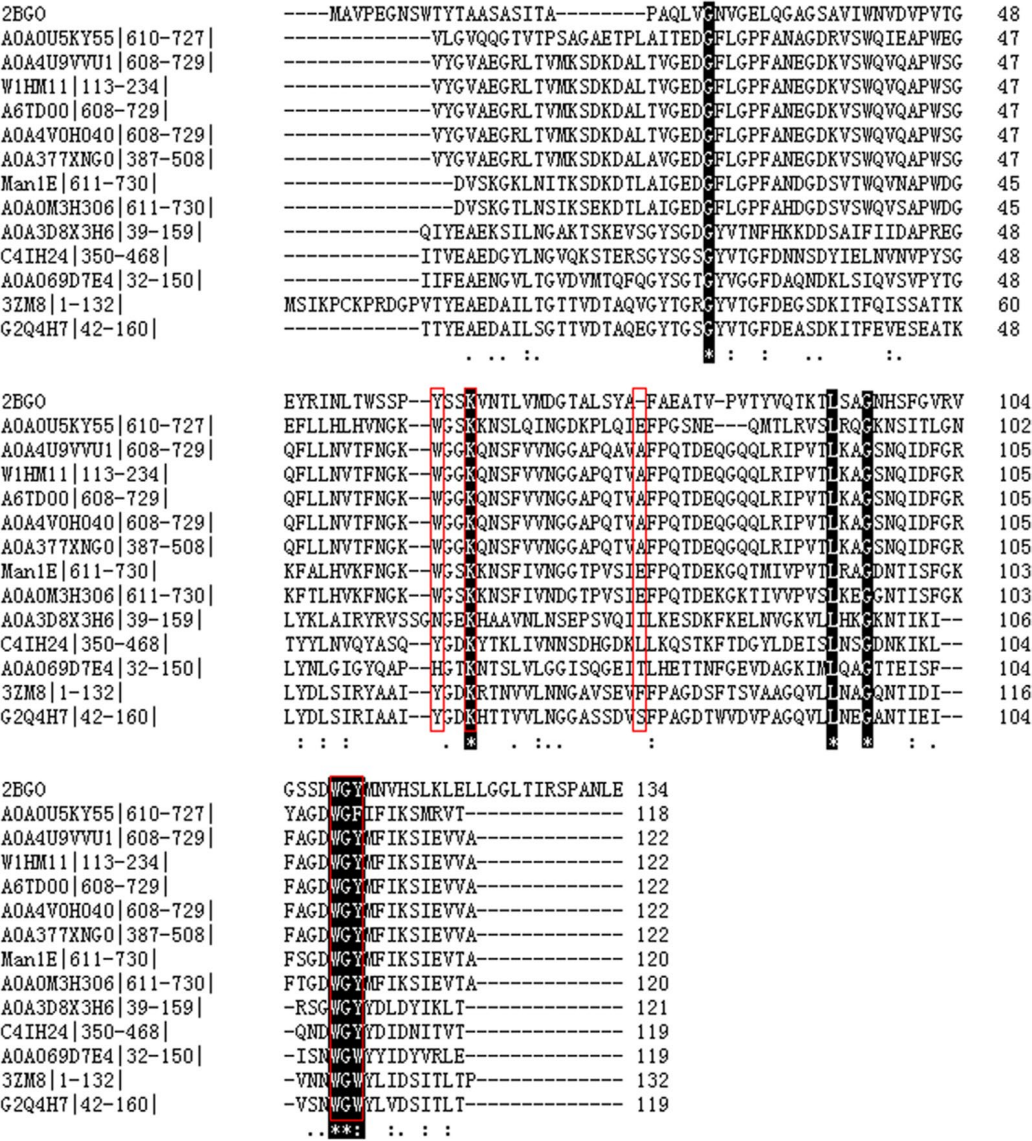
Multiple sequence alignment of CBMs of Endo-β-1,4-mannanase. Man1E from *Enterobacter aerogenes* B19 (this study), Endo-β-1,4-mannanases from *Cellvibrio japonicus* (PDB code, 2BGO), *Podospora Anserina* (PDB code, 3ZM8), *Klebsiella pneumoniae* IS39 (Uniprot Entry, W1HM11), *Klebsiella pneumoniae subsp. pneumoniae* strain ATCC 700721 (Uniprot Entry, A6TD00), *Erwinia gerundensis* (Uniprot Entry, A0A0U5KY55), *Myceliophthora thermophila* strain ATCC 42464 (Uniprot Entry, G2Q4H7), *Paenibacillus* sp. TCA20 (Uniprot Entry, A0A069D7E4), *Clostridium butyricum* E4 str. BoNT EBL5262 (Uniprot Entry, C4IH24), *Bacillus megaterium* (Uniprot Entry, A0A3D8X3H6), *Klebsiella pneumoniae* (Uniprot Entry, A0A377XNG0), *Escherichia coli* (Uniprot Entry, A0A4V0H040), *Enterobacter aerogenes* (Uniprot Entry, A0A0M3H306), *Enterobacter aerogenes* (Uniprot Entry, A0A4U9VVU1). Conserved residues were expressed in reverse colour, residues participating likely in the substrate binding were marked with red box

Sequence alignments of Man1ECBM, 11 CBM6s and 2 CBM35s (2BGO and 3ZM8|1-132|) showed that 3 glycines (G), 1 lysine (K), 1 Leucine (L) and 1 tryptophan (W) were highly conservative in all CBMs. The six amino acid residues were located at positions 23, 60, 92, 95, 108 and 109 in Man1ECBM (Fig. 1). According to the literature [36], there is a hydrophobic platform, which consists of three aromatic amino acid residues Phe,Trp and Trp, participating in substrate binding. In CtCBM35 and PaCBM35 (PDB ID: 2BGO), these three residues are Phe100, Trp129,Try131, and Phe87,Trp117, Trp119, respectively. However no such a hydrophobic platform existed in Man1ECBM where Glu75 replaced Phe. Through the analysis for multiple sequence alignment, the characteristic sequence, “Trp-Gly-Tyr (WGY)” motif, was found in Man1ECBM. The WGY motif, which may determine the specificity of mannose binding, consists generally in CBM35 and CBM6 [36]. The Tyrosine residue at the third position of WGY motif is relatively conservative, which can be replaced with Trp or Phe. For example, it was found to be Trp129-Gly130-Tyr131 in CtCBM35 and Trp107-Gly108-Tyr109 in Man1ECBM, but Trp117-Gly118-Trp119 in PaCBM35 (PDB ID: 3ZM8) (Fig. 1). Trp57 and Lys60 in Man1ECBM corresponded to Tyr80 and Lys83 in CtCBM35, and Tyr60 and Lys63 in PaCBM35, which have been proven to participate in the substrate binding [36].

### The advanced structural analysis of Man1E

The secondary structure of Man1E analyzed by PSIPRED program showed that it included 20.11% Helix (147aa), 23.26%, Strand (170aa), and 56.63% Coil. The amino acid sequence of Man1E was analyzed with NCBI CDD Tool (https://www.ncbi.nlm.nih.gov/cdd) and two CDD matches to Man1E were found, including Glycosyl hydrolase family 1 (116 aa–14 aa) and CBM6-CBM35-CBM36_like family (611 aa–730 aa) (Additional file 2: Figure S3). The result indicated that Man1E belonged to GH1 family. CBM6-CBM35-CBM36_like family contain carbohydrate binding module family 6 (CBM6), also described as cellulose binding domain family VI, and associated CBMs (CBM35 and CBM36). These CBMs without catalytic function have been found in a series of enzymes. They demonstrate activities on a variety of carbohydrate substrates, such as cellulose, xylan, β-glucan, mannan, agarose, and araban. These domains promote the closer binding of additional catalytic modules with their specific, insoluble substrates. A number of CBMs are related to the domains of glycoside hydrolase (https://www.ncbi.nlm.nih.gov/Structure/cdd/cddsrv.cgi). According to the similarity of amino acid sequences, three-dimensional structures and catalytic mechanism, β-mannanases can be divided into three families: GH5, GH26 and GH113 [1]. But Man1E in this study was found belonging to CH1 family, different from all reported β-mannanases.

**Fig. 2:**
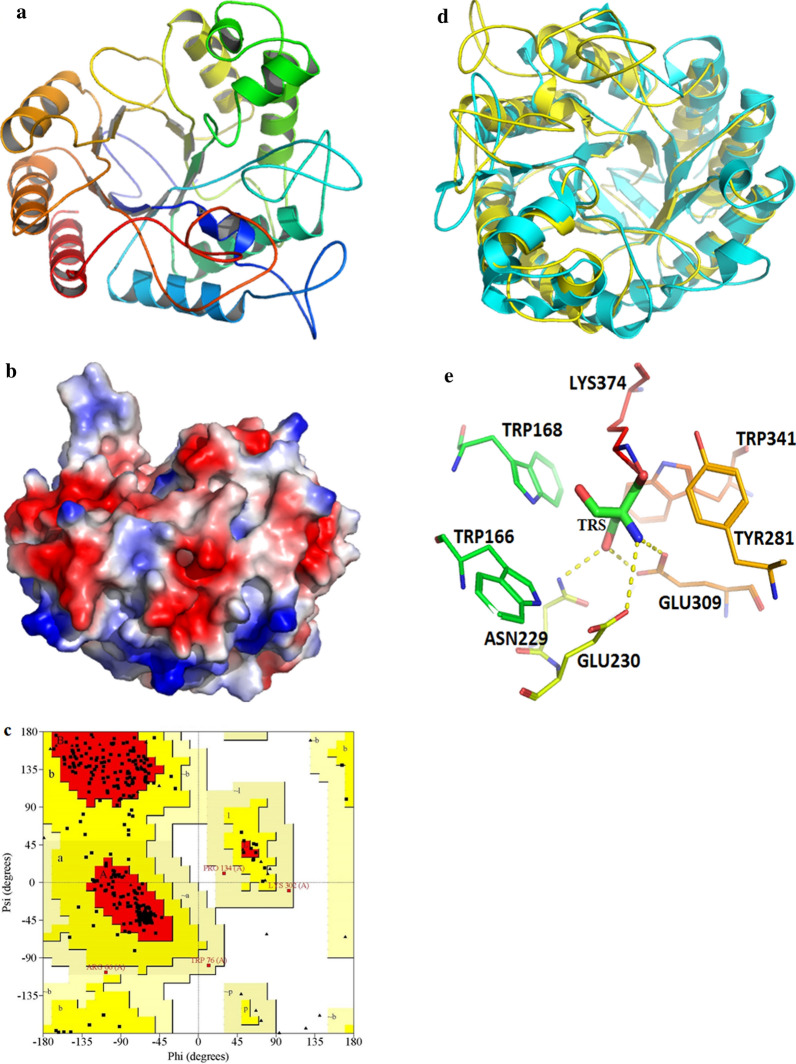
Homology modeling and the active center of Man1E from *Enterobacter aerogenes* B19. **a** The modelled 3D-structure of Man1E based on *Rhizomucor miehei* Man5A. Blue indicated N- terminus, red indicated C- terminus. **b** Vacuum electrostatic surface of Man1E. **c** Ramachandran plot of Man1E homologous model structure. Red area indicated most favoured regions, yellow area indicated additional allowed regions, faint yellow area indicated generously allowed regions, white area indicated disallowed regions. **d-e** The active center of Man1E predicted by bSiteFinder. Overlap of Man1E and *Pa*Man5A (PDB code, 3ZIZ) (**d**). yellow represents Man1E, cyan represents *Pa*Man5A. The amino acid residues linked to the ligand in the predicted catalytic center (**e**). TRS represents 2-amino-2-hydroxymethyl-propane-1,3-diol

The structural model of Man1E was established with SWISS-MODEL server, based on the structures of *R. miehei* Man5A (PDB code, 4lyp.1.A) [35]. The resultant structure was represented in the Fig. 2a. The rationality of the structure models of Man1E was assessed with SAVES v5.0 tool ((https://servicesn.mbi.ucla.edu/SAVES/), the Ramachandran plots were drawn using the PROCHECK program (Fig. 2b). For the model, there were 85.1% of total residues (367) in most favoured regions, 14.0% residues in additional allowed regions, 0.9% residues in generously allowed regions and no residues in disallowed regions. The percentage of total residues (367) in most favoured regions is close to 90%, which is the standard for a good quality model [37]. The result indicated that the model were reasonable and reliable. The overall structure of Man1E showed the typical (α/β)_8_-barrel motif, like β-mannanases of GH5 and GH26 families [38]. For the surface electrostatic potential, negative charges were a little more than positive charges, which was in accordance with isoelectric point of partial acidity (5.91) of the enzyme. The 3D structure of Man1E contained ten α-helixes, eight β-strands and a number of loops. The four loops that linked α1 and α2, β2 and α3, β5 and β6, and β8 andα11 were composed of 20–45 amino acid residues, whereas the other four loops that linked β3 and α4, β4 and α7, β6 and α8, and β7 and α9 consisted of 10–12 amino acid residues. These loops indicated that the catalytic domin of Man1E possessed considerable flexibility. In addition, a obvious loop at the N-terminus was located at the bottom of the barrel in Man1E, different from *T. fusca* β-mannanase and *L. esculentum* β-mannanase which contain two short β-strands at the bottom of the barrel [16, 34].

### The catalytic module of Man1E

The catalytic center was predicted by bSiteFinder (http://binfo.shmtu.edu.cn/bsitefinder/) using *Pa*Man5A (PDB code, 3ZIZ) from *Podospora anserine* as a template, the overlap of the two β-mannases was shown in Fig. 2d. The result showed that several highly conserved regions existed between Man1E and *Pa*Man5A (Fig. 2d). The catalytic elements contained a platform composed of four hydrophobic residues TRP166, TRP168, TYR281 and TRP341, which were exposed to solvent, and four charged hydrophilic residues Asn229, Glu230, Glu309 and Lys374 (Fig. 2e). TRS docking into the catalytic module of Man1E showed the same result as predicted by bSiteFinder (Additional file 1: Figure S1e). The acid–base residue and nucleophilic residue in β-mannanses of CH5 and CH26 families are generally conserved and glutamate (Glu). For example, in BsMan26A (CH26), BCman (CH26), *Pa*Man5A (CH5) and RmMan5A(CH5) the acid–base residue and nucleophilic residue are Glu176 and Glu275 [39], Glu167and Glu266 [40], Glu177 and Glu283 [41], Glu175 and Glu293 [42], respectively. Combined with Fig. 1, the acid–base residue and nucleophilic residue in Man1E were speculated to be Glu230 and Glu309, respectively. Asn229 formed hydrogen bond with the ligand through carbonyl oxygen in the side chain. Glu230 and Glu309 linked to the ligand by hydrogen bond through hydroxyl group of carboxyl group, Glu309 also constituted hydrogen bond with the ligand through carbonyl oxygen of carboxyl group. Lys374 was combined with the ligand through amino-group of the side chain.

**Fig. 3:**
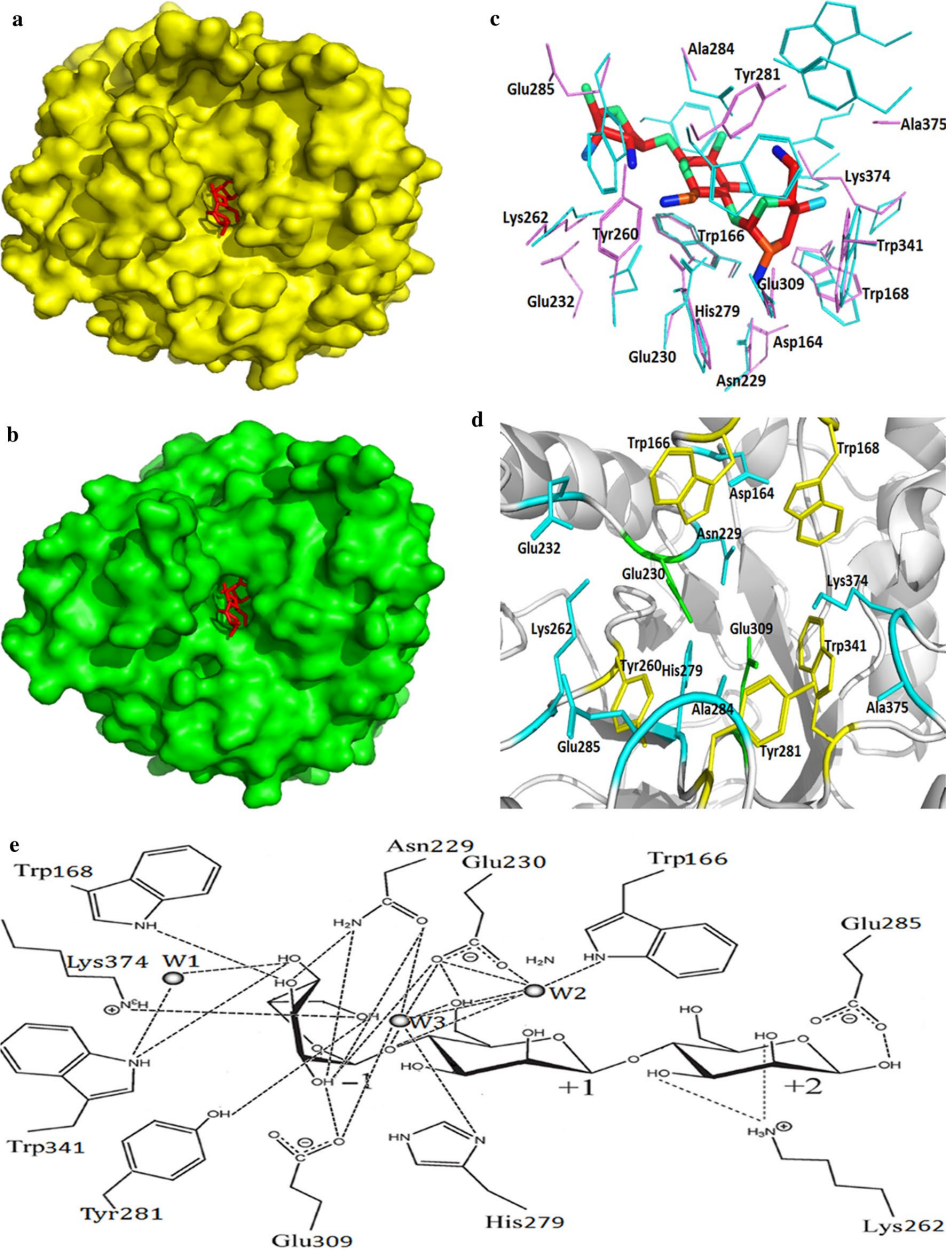
The prediction and analysis of the catalytic domin performed by structural superposition. **a** The surface view of Man1E docking mannotriose, red stick represented mannotriose. **b** The surface view of RmMan5B (PDB code, 4LYP) from *Rhizomucor miehei* docking mannotriose, red stick represented mannotriose. **c** Structural superposition of amino acid residues in the active domain of Man1E and RmMan5B-mannotriose, violet represented Man1E, cyan represented RmMan5B. **d** Amino acid residues in the catalytic module of the complex of Man1E–mannotriose, green represented the acid–base residue and the nucleophilic residue, yellow represented hydrophobic residues, cyan represented other residues. All figures were created by PyMOL 2.3.2. **e** Diagrammatic sketch of hydrogen bond interactions between mannotriose and amino acid residues at subsites − 1 to + 2. W1, W2, W3 represented water molecules. The figure were drown using ChemDraw software

Like RmMan5B, the active region of Man1E took on a deep slot-like pocket (Fig. 3a, b). Superposition of amino acid residues in the active domain of Man1E and RmMan5B-mannotriose was shown in Fig. 5c. At the − 1 and + 1 subsites, seven superimposed residues were found between RmMan5B and Man1E. In Man1E, these residues included Trp166, Trp168, Asn229, Glu230, His279, Glu309 and Trp341. At the -2 subsite, only Lys residue was found to be superimposed (Lys262 in Man1E). In GH family 5 enzyme structures, there are eight highly conserved residues at the −1 and + 1 subsites [16]. Six out of these eight highly conserved residues were found in the β-mannanases (Figs. 1 and 3c; Trp166, Asn229, Glu230, His279, Glu309 and Trp341 in Man1E). The catalytic region of Man1E-mannotriose possessed a hydrophobic platform extending from the − 1 subsite to the + 1 subsite (Fig. 3d) which consisted of five aromatic amino acid residues. These hydrophobic residues included Trp166, Trp168, Tyr260, Tyr281 and Trp341. Trp341 had the same plane orientation as the pyranose sugar at the − 1 subsite, Trp166 and the pyranose sugar were at the + 1 subsite are in plane direction (Fig. 3c). The similar hydrophobic platform has been found in bacterial GH5 family β-mannanases and GH26 family β-mannanases. For example, the hydrophobic platform in RmMan5B is composed of Trp117, Trp119, Trp257, Trp261, Trp384 and Tyr385, whereas in GH26 family BCman it contains Trp302, Trp298, Trp172, and Trp72 [40]. All of the residues located in the catalytic region of Man1E included Ala375, Lys374, Trp341, Glu309, Glu285, Ala284, Tyr281, His279, Lys262, Tyr260, Glu232, Glu230, Asn229, Trp168, Trp166 and Asp164 (Fig. 3d). The acid–base catalytic glutamate Glu230 and the nucleophilic glutamate Glu309 lay at the loop between β-strand 4 and α-helix 7, and at the loop between β-strand 7 and α-helix 9, respectively. Except for Ala375, Ala284, Tyr260, Glu232 and Asp164, All other residues in the catalytic region of Man1E formed hydrogen bonds directly or indirectly with the substrate (Fig. 3e). At the − 1 subsite, a number of hydrogen bonds were observed. At the − 1 subsite, the acylamino oxygen of Asn229, carboxyl oxygens of Glu230 and Glu309 produced hydrogen bonds with O2 of the sugar. Asn229 also linked to O2 and O3 of the sugar through the amido of acylamino. The indole nitrogens of Trp168 and Trp341 made a polar contact with O3 and O4 of the sugar, respectively. Lys374 and Tyr281 connected with O6 of the sugar through amido and hydroxyl of side chains, respectively. At the + 1 subsite, the acid–base catalytic residue Glu230 generated hydrogen bond with O6 of the sugar. Glu230 also indirectly interacted with Trp166, Asn229, His279 and Glu309 by forming hydrogen bonds with water molecules. The indole nitrogen of Trp166 and the carboxyl oxygen of Glu230 indirectly acted on oxygen of the glucosidic bond through a water molecule (W2). The acylamino oxygen of Asn229, carboxyl oxygen of Glu230, imidazole nitrogen of His279 and carboxyl oxygen of Glu309 indirectly acted on oxygen of the glucosidic bond through a water molecule (W3). At the + 2 subsite, Lys262 formed hydrogen bond with O2 and O3 of the sugar through the amido of side chain. Gu285 was linked to O1 of the sugar by hydrogen bond through the carboxyl of side chain.

**Fig. 4:**
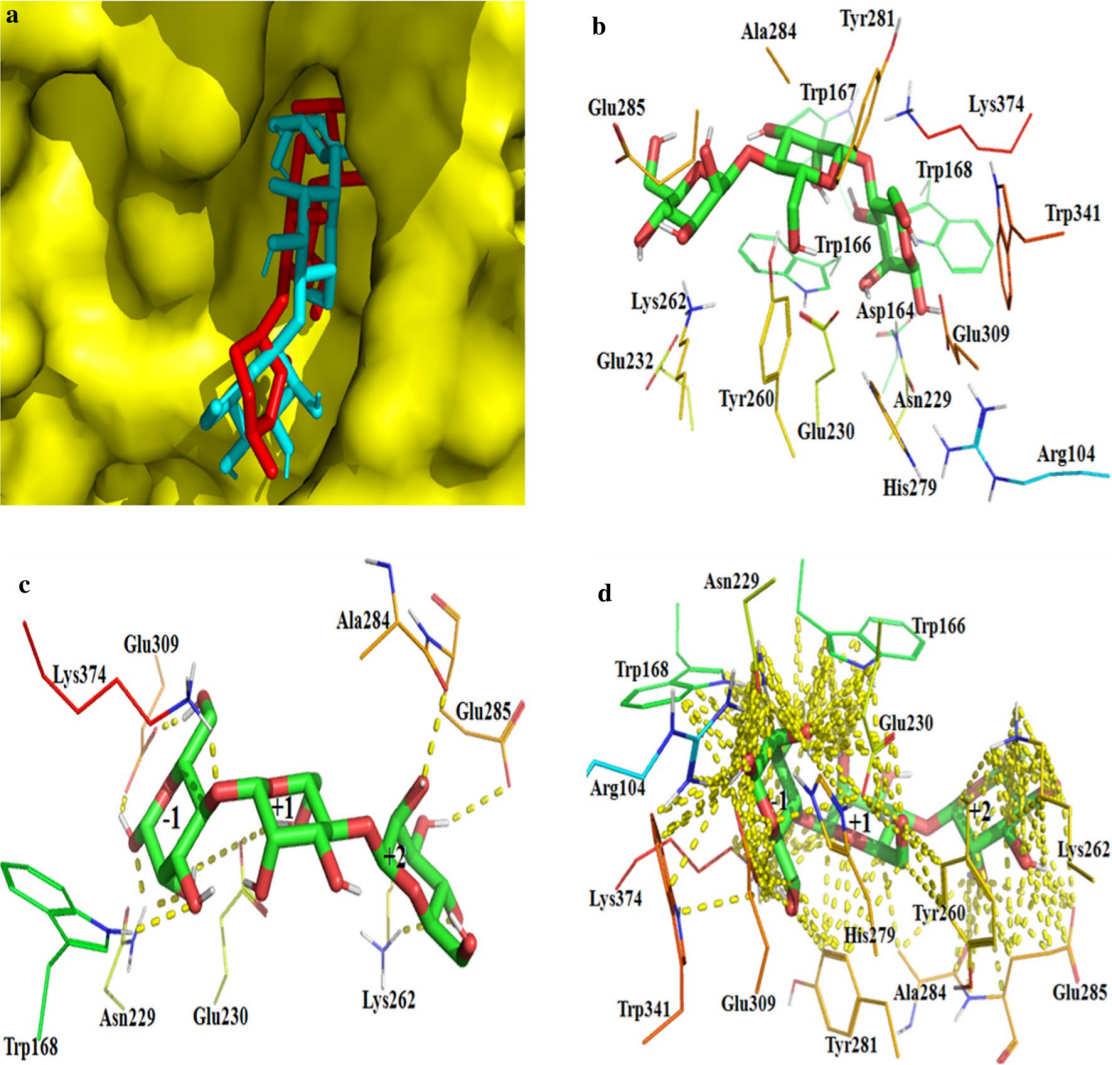
The analysis of the catalytic domin performed by molecular docking. **a** The comparison of two conformations of mannotriose, red stick represented the conformation from RmMan5B-mannotriose complex, cyan stick represented the conformation from the molecular docking between Man1E and mannotriose. **b** Amino acid residues in the catalytic module of Man1E-mannotriose complex. **c** Diagrammatic sketch of polar contacts between mannotriose and amino acid residues at subsites − 1 to + 2. **d** Diagrammatic sketch of any contacts (within 4 Å) between mannotriose and amino acid residues at subsites − 1 to + 2

Docking analysis can provide very useful information on the key residues involved in the interactions between enzyme and substrate. The noncovalent interactions between the ligand and receptor are important for investigating the subsite of enzyme–substrate binding before the catalytic reaction [43]. In order to reveal the binding subsites and involved residues, molecule docking was performed using mannotriose as the ligand. The docking result showed that mannotriose was properly positioned in the catalytic cavity, thus forming a stable complex with the enzyme. But the conformation of mannotriose produced by molecular docking was obviously different from that obtained by structural superposition (Fig. 4a). The amino acid residues surrounding mannotriose included Arg104, Asp164, Trp166, Trp167, Trp168, Asn229, Glu230, Glu232, Tyr260, Lys262, His279, Tyr281, Ala284, Glu285, Glu309, Trp341 and Lys374 (Fig. 4b). The possible residues involved in substrate binding and catalysis were analyzed from the perspective of polar interactions. As shown in Fig. 4c, there were eight residues, including Trp168, Asn229, Glu230, Lys262, Ala284, Glu285, Glu309 and Lys374, formed hydrogen bonds with mannotriose from − 1 subsite to + 2 subsite (Fig. 4c). Compared with structural superposition, the residues involved in the formation of hydrogen bonds did not include Trp166, His279, Tyr281 and Trp341, but additional Ala284 participated in the formation of hydrogen bond (Figs. 3e and 4c). The eight residues mentioned above not only formed hydrogen bond with mannotriose, but also produced nonpolar contacts. In addition, a large number of nonpolar interactions were formed between Arg104, Trp166, Tyr260, His279, Tyr281, Trp341 and manotriose (Fig. 4d). As could be seen from Fig. 4d, a hydrophobic platform, which was composed of Trp166, Trp168, Tyr260, Tyr281 and Trp341, existed in the catalytic module of Man1E.

### The carbohydrate binding module of Man1E

**Fig. 5:**
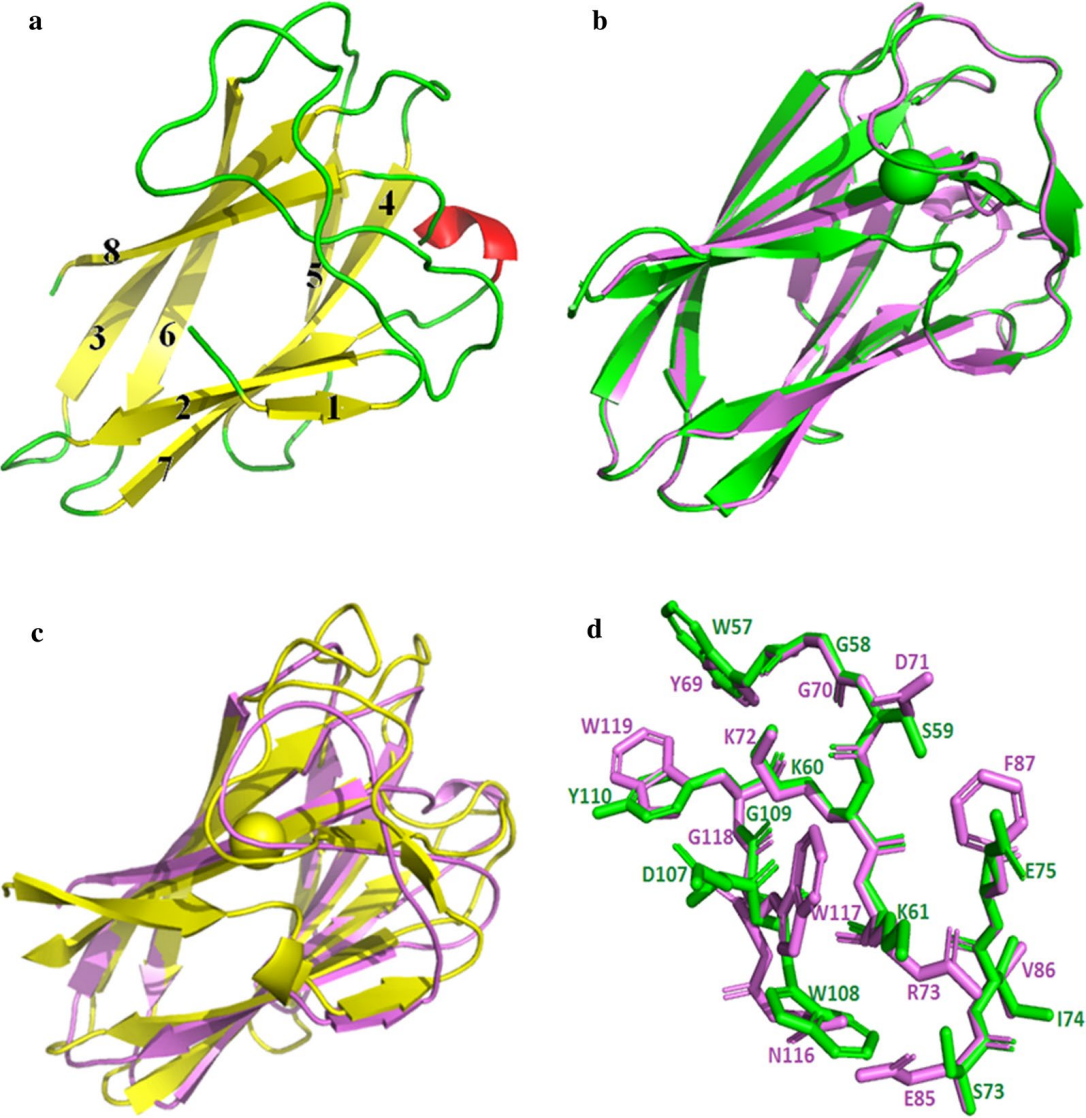
The structural prediction of Man1ECBM and the analysis performed by structural superposition. **a** Man1ECBM; green represented loop, yellow represented β-strand, red represented α-helix. **b** Superposition of Man1ECBM (*violet*) and *Pa*CBM35 domain (*green*). the *green sphere* represented calcium ion. **c** Superposition of Man1ECBM (*violet*) and CtCBM35*(yellow*) from. a GH26 *β*-mannanase of *thermocellum*. the *yellow sphere* represented calcium ion. **d** Overlap of the amino acid residues that might bind to substrate in Man1ECBM (green) and *Pa*CBM35 (*violet*)

Man1E possessed a noncatalytic carbohydrate binding module (CBM) at its C-terminal. Noncatalytic CBMs exist in most of glycoside hydrolases. When glycoside hydrolases attack generally unapproachable macromolecular substrates, CBMs may increase the concentration of the additional enzymes surrounding the substrate, thus getting involved in a twofold to fivefold increase in the activity of endocutting enzymes [44]. The overall structure of Man1ECBM was shown in Fig. 5a. It was composed of four pairs of antiparallel β-strands, a short α-helix and several loops. The loop between β1 and β2 contained 27 amino acid residues, other loops between two β-strands or between α-helix and β-strand consisted of 5–8 residues. The eight β-strands formed a hydrophobic cavity in which a number of Val, Phe and Ile residues were located. The homology structure of ManE1CBM was *Pa*CBM35. The main differences between Man1ECBM and *Pa*CBM35 were that *Pa*CBM35 harbored eleven β-strands and no α-helix (Fig. 5b). Man1ECBM also diaplayed higher homology in structure with *C. thermocellum* CBM35 (PDB code 2W1W) (Fig. 5c). Three additional β-strands and the different length and quantity of the loops occurred in ManE1CBM compared with *C. thermocellum* CBM35. Calcium ion was not found in ManE1CBM, but both *Pa*CBM35 and *C. thermocellum* CBM35 bind to a calcium ion (Fig. 5b, c). Overlap of Man1ECBM and *Pa*CBM35 showed that 11 amino acid residues formed a “Bending Channel”, in which substrates were likely to be bound. These residues included Trp57, Gly58, Ser59, Lys60, Lys61, Ser73, Ile74, Asp107, Try108, Gly109 and Tyr110, containing the WGY motif (Fig. 5d).

**Fig. 6:**
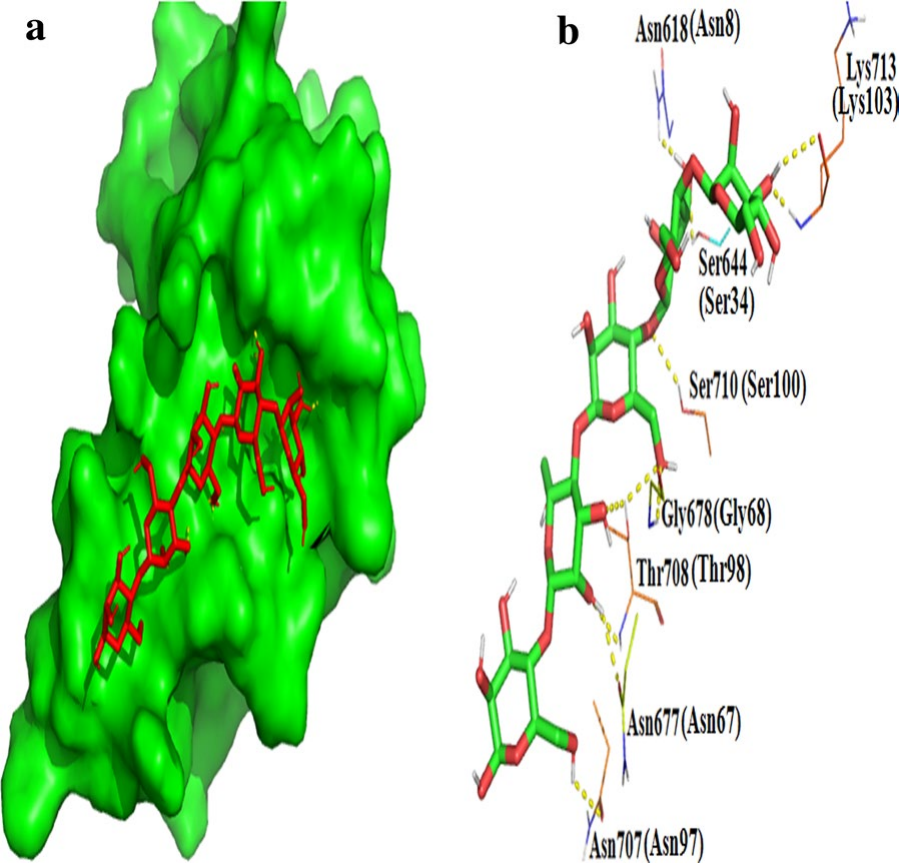
The analysis of Man1ECBM performed by molecular docking. **a** The surface view of Man1ECBM docking mannotriose, red stick represented mannopentaose. **b** Diagrammatic sketch of polar contacts between Man1ECBM and mannopentaose. Residue numbers out of brackets represented the residue order in the complete *β*-mannanase. Residue numbers in brackets represented the residue order in Man1ECBM

CBMs are divided into more than 50 families based on sequence in cazy database [45]. Because of their modular features, they are often studied independently [46]. In order to investigate the residues involved in binding substrate, molecule docking was conducted using mannopentaose as the ligand. The docking result showed that mannopentaose was appropriately located in a groove in the surface of Man1ECBM (Fig. 6a). The possible residues involved in substrate binding included eight residues, namely, Asn8, Ser34, Asn67, Gly68, Asn97, Thr98, Ser100 and Lys103 (Fig. 6b). The result of molecular docking was quite different from that of superposition analysis (Figs. 5d and 6b). The reason for this difference may be explained that CBM35 undergoes significant conformational change upon ligand binding, and the binding specificity may mainly depend on the curvature of the binding site and the size of the binding groove [47]. Whereas, in molecular docking procedures, the receptor molecules are assumed to be rigid. It is likely a more accurate method to determine the binding residues by comparing the surfaces of the free and bound structures of CBM35 with magnitude of the chemical shift changes from the substrate [47].

### Gene expression in *Escherichia coli*

**Fig. 7:**
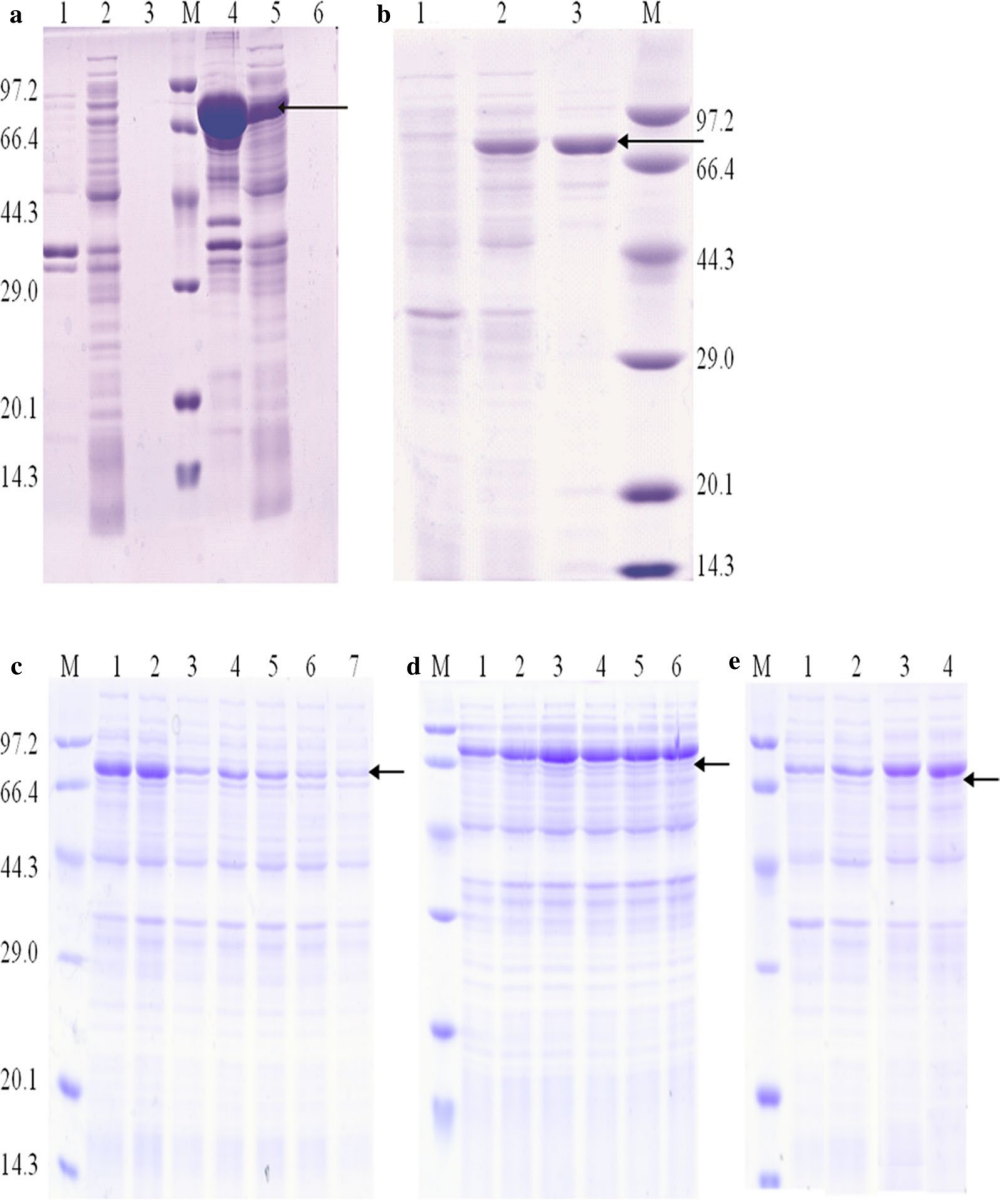
The expression and purification of recombianant Man1E analyzed by SDS-PAGE. **a** Recombinant Man1E analyzed by SDS-PAGE. Lane 1: Precipitation after cell lysis from Escherichia coli BL21(DE3) harboring pET28a (+); Lane 2: Cell lysate from Escherichia coli BL21(DE3) harboring pET28a (+); Lane 3: Fermentation supernatant from *Escherichia coli* BL21(DE3) harboring pET28a (+); Lane M:Marker; Lane 4: Precipitation after cell lysis from *Escherichia coli* BL21(DE3) harboring pET28a (+)-*Man1E*; Lane 5: Cell lysate from *Escherichia coli* BL21(DE3) harboring pET28a (+)-*Man1E*; Lane 6: Fermentation supernatant from *Escherichia coli* BL21(DE3) harboring pET28a (+)-*Man1E*. **b** SDS–PAGE analysis of purified Man1E. Lane 1: Supernatant after cell breakage from *Escherichia coli* BL21(DE3) harboring pET28a (+); Lane 2: Supernatant after cell breakage from *Escherichia coli* BL21(DE3) harboring pET28a (+)-*Man1E*; Lane 3: Purified recombinanant Man1E; Lane M: Marker. **c-e** Optimization of soluble expression for recombianant Man1E. Induction temperature (**c**). Lane M: Marker; Lane 1: 16 °C; Lane 2: 20 °C; Lane 3: 24 °C; Lane 4: 28 °C; Lane 5: 32 °C; Lane 6: 37 °C; Lane 7: 42 °C. PTG concentration (**d**). Lane M: Marker; Lane 1: 0.2 mM; Lane 2: 0.4 mM; Lane 3: 0.6 mM; Lane 4: 0.8 mM; Lane 5: 1.0 mM; Lane 6: 1.2 mM. Induction time (**c**). Lane 1: 4 h; Lane 2: 8 h; Lane 3: 12 h; Lane 4: 16 h. The band shown by the arrow was recombinant Man1E

The appropriate *E. coli* BL21(DE3) harboring pET-28a(+) –*Man1E* strains were inoculated in LB medium and cultured for 24 h at 37 °C, 200 rpm in shake flasks. The cells and the supernatant were collected separately for β-mannanase assays. Comparative analysis for the proteins of the *E. coli* BL21(DE3) host strains and the recombinant strains by SDS-PAGE displayed that a number of the recombinant proteins existed in insoluble form (Fig. 7a, Lane 4) and some in an soluble form (Fig. 7a, Lane 5) in the cells. The molecular weight of recombinant β-mannanase was about 82.5 kDa, which was corresponding to the predicted value of Man1E (Fig. 7a). It is reported that the Mws of microbial β mannanase are between 17.7 and 130 kDa [27, 48–50]. For example, the Mws of β-mannanases are 39–40 kDa from *B. subtilis* KU-1 [51], 38.04 kDa from *Bacillus* sp. MK-2 [52], 45 kDa from *Klebsiella pneumoniae* SS11 [53], 49 kDa from *Vibrio* sp. MA-138 [54], 66 kDa from alkaliphilic *Bacillus* sp. [55]. Only a few literatures have reported β-mannanases from *Klebsiella*-*Enterobacter* group, which possess the Mws with 43 kDa, 45 kDa, 90 kDa (shown in Table 1). 82.5 kDa of β-mannanase in this study was reported for the first time. The single factor experiment was used for investigating the effect of different factors on soluble expression of the recombinant β-mannanase.

**Table 1:** Physicochemical properties of characterized β-mannanases from *Klebsiella*-*Enterobacter* group strains

Bacteria strain	Mw (kDa)	Optimum pH	Optimum Temp (°C)	Stability pH	Stability Temp (°C)	Substrate preferred	K_m_ (mg/mL)	V_max_	References
*Enterobacter aerogenes* B19	82	6.5	55	3.5–9.0	≤ 60	LBG	3.01	854.22 ± 3.85 μmol/(mL min)	This work
*Klebsiella pneumoniae* SS11	45	9.0	70	7.0–10.6	≤ 70	LBG	1.66	833.33 μmol/(mL min)	[53]
*Klebsiella oxytoca* KUB-CW2-3	43	4–6	40	4–10	≤ 40	KGM	1.038	6.183 μmol/(mL min)	[63, 65]
*Enterobacter* sp. N18	90	7.5	50	3.0–10.0	≤ 60	LBG	3.427	134.05 μmol/(mg min)	[62]
*Enterobacter ludwigii* MY271	43	7.0	55	2.0–10.0	≤ 60	KGM	18.8 ± 0.8	2481 ± 13 μmol/(mg min)	[59]
*Enterobacter asburiae* SD26	–	6.0	50	–	–	LBG	25	2500 μmol/(mL min)	[69]

The total soluble expression of Man1E reached maximum at 20 °C (Fig. 7c), the mannanase activity was 608.11 U/mL. High induction temperatures displayed negative effects on the accumulation of soluble β-mannanase. The soluble expression and activity of Man1E increased with the increase of IPTG concentration in a range of 0.2–0.6 mM, the mannanase activity was 720.81 U/mL at 0.6 mM IPTG (Fig. 7d). Man1E presented higher expression after IPTG induction for16 h (Fig. 7e), the mannanase activity was 642.25 U/mL, however, there was no significant difference in soluble expression and activity between 12 and 16 h. By comparison, the optimal inductive temperature and time for Man1E gene expression similar to those of β-mannanase genes from *Bacillus circulans* NT 6.7 (for 16 h at 18 °C) [56] and *Pantoea agglomerans* A021 (for 15 h at 18 °C) [57]. However, for the expression of three β mannanase genes, the IPTG concentrations were different, which were 0.6 mm, 1.0 mm and 0.05–0.15 mm, respectively.

### The purification and Biochemical characterization of Man1E

The crude extract of intracellular proteins from the recombinant strains was applied to affinity chromatography on a Ni–NTA column. The eluant was analyzed on SDS-PAGE gel and the result proved that the enzyme could achieve electrophoretic purity through this one-step purification scheme, and the Mw value of the purified β-mannanase was about 82.5 kDa (Fig. 7b), which was accordance with the expected value.

**Fig. 8:**
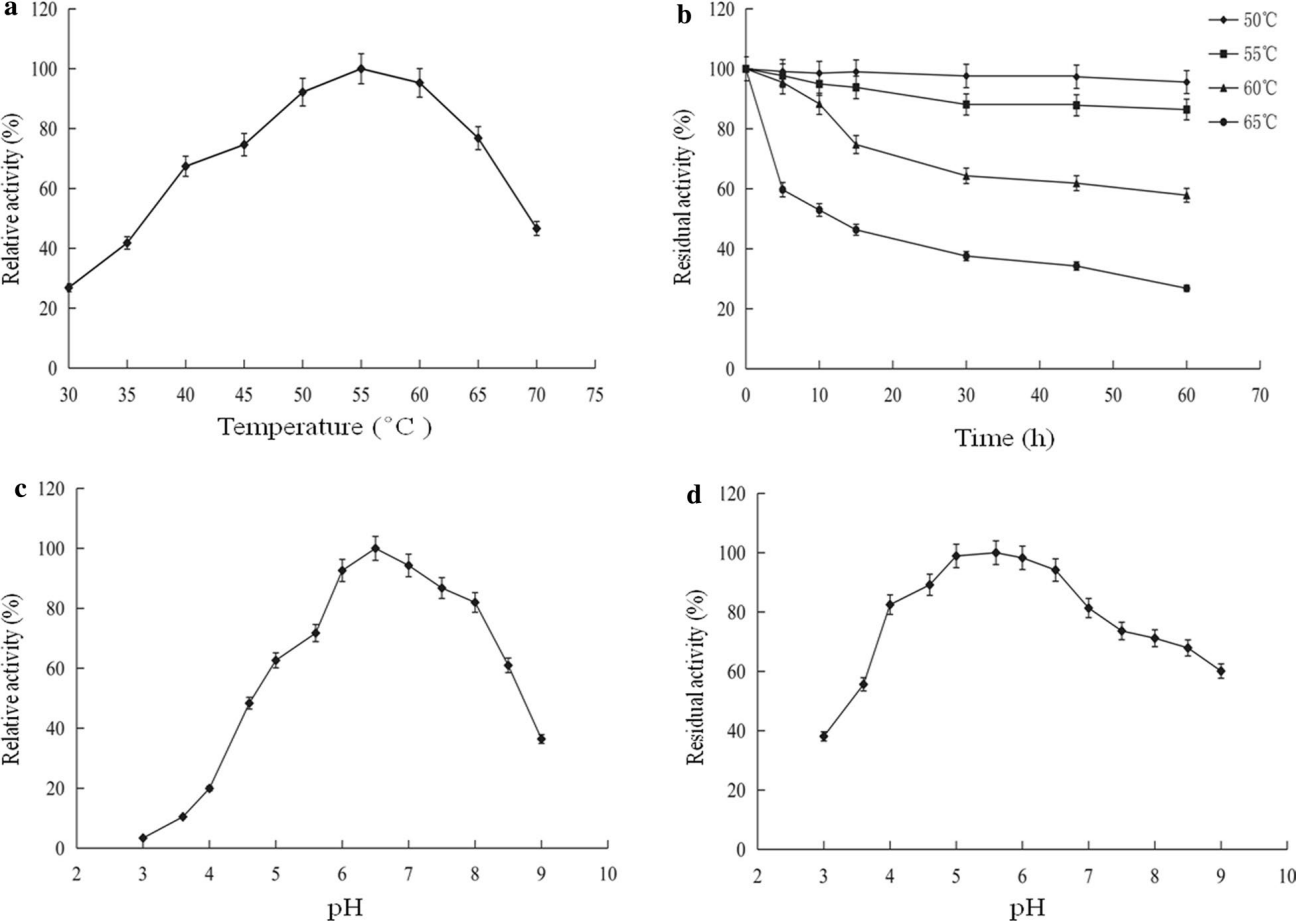
Effects of temperature and pH on activity and stability of recombinant β-mannanase. **a** Optimum temperature. **b** Thermostability. **c** Optimum pH. **d** pH stability

The purified Man1E was incubated in a temperature range of 30 °C to 70 °C to determine the effect of temperature on β-mannanase activity. Man1E performed the highest activity at 55 °C. The β-mannanase activity increased gradually from 30 to 55 °C, but displayed a decreasing trend when the temperature was above 55 °C (Fig. 8a). Reportedly, the optimum temperature of β mannanases from different strains varied from 45 to 85 °C [3]. For instance, Man5 from *Thermotoga maritime* displays the optimum temperature of 90 °C, which is the highest temperature reported so far [58]. Whereas the optimal temperature of β mannanases from *E. ludwigii* MY271 [59], *B. subtilis* YH12 [60], *B. subtilis* NM-39 [61] was 55 °C, the same as that of Man1E. The optimum temperature value of Man1E is close to those of β-mannanases from *Enterobacter* sp. strain N18 [62] and *Klebsiella oxytoca* CW23 [63]. The purified Mann1E was stable below 60 °C, the enzyme still remained above 60% activity after being incubated for 1 h at 60 °C, especially below 55 °C retained more than 85% of activity after being incubated for 1 h (Fig. 8b), which shows similarity to the relevant enzymes from *E. ludwigii* MY271 and *Penicillium occitanis* Pol6 [64], and inferior to the corresponding enzymes from *Enterobacter* sp. strain N18 and *K. pneumoniae* SS11. However, The thermal stability of purified Mann1E was better than β-mannanases from *K. oxytoca* KUB-CW2-3 [65] and *Aspergillus sulphureusis* [66].

The activity determination at pH values in the range of 3.0–9.0 showed that Man1E exhibited the maximal activity at pH 6.5. Below or above the optimum pH, the activity declined to about 21.3% at pH 4.0 and 37.5% at pH 9.0 (Fig. 8c). It has been reported that the optimal pH value of β-mannanases from *B. subtilis* YH12, *B. subtilis* G1 [67], *Bacillus stearothermophilus* [68] is 6.5, which is the same as that of Man1E, whereas β-mannanases from *E. ludwigii* MY271 and *K. oxytoca* CW23 [65] show the highest activity at neutral pH. β-mannanases from *Enterobacter* sp. strain N18, *K. pneumoniae* SS11 and *Enterobacter asburiae* SD26 [69] show optimum catalytic capacity at pH 7.5, 9.0 and 6.0, respectively. In addition, it is known that microbial β-mannanases have the maximum activity in a wide pH range of 4.0 to 10.0 [70]. Man1E was stable over a wide pH range, it remained more than 60% activity at pH 4.0–9.0, remarkably more than 80% activity at pH 4.0–7.0. Even at low pH values of 3.0 and 3.5, Man1E still displayed 38.1% and 55.3% activity respectively (Fig. 8d). The pH stability of Man1E is similar to the reported β-mannanase from *Klebsiella*-*Enterobacter* group strains (Table 1). The good pH stability of Man1E makes it attractive for industrial applications, for example, it can still function as feed additive in animal gastrointestinal tract and be used in pulp bleaching.

The effects of different metal ions on Man1E activity were shown in Table 2. Compared with the control sample without any metal ions, the purified Man1E activity was activated by Co^2+^, Mn^2+^, Zn^2+^, Ba^2+^ and Ca^2+^. The presence of 2 mM Co^2+^ and Mn^2+^ could increase the activity by 35.6% and 26.9% respectively. However, the enzyme activity was significantly inhibited by 2 mM Cu^2+^ that decreased the relative activity by 34.7%. The enzyme activity was moderately inhibited by K^+^ and Mg^2+^. The enzyme activity showed little change in the presence of Na^+^, Ni^2+^ or Fe^2+^. The activation of Co^2+^, Ba^2+^ and Ca^2+^ on Man1E activity is similar to the β-mannanase from *K. pneumoniae* SS11, but the effects of Zn^2+^, Mg^2+^ Na^+^, Ni^2+^ and Fe^2+^ are completely different between the two β-mannanases. Mn^2+^, Co^2+^, Ca^2+^ and Ba^2+^ could enhance the enzyme activity of Man1E and β-mannanase from *Paenibacillus* sp. CH-3 [71], but Mg^2+^ and Zn^2+^ just exhibit the opposite effects on the two enzymes. Cu^2+^ exerts a strong inhibitory effect on many β-mannanases, for example, the β-mannanases from *E. ludwigii* MY271, *B. circulans* NT 6.7 [72], *Paenibacillus cookie* [73] and *Bacillus* sp. MK-2.

**Table 2:** Effect of various metal ions on the activity of recombinant Man1E

Metal ions	Relative enzyme activity
CK	100
Na^+^	101.25 ± 2.05
K^+^	83.61 ± 0.34
Mn^2+^	126.87 ± 3.07
Mg^2+^	91.09 ± 2.64
Zn^2+^	109.67 ± 0.39
Ca^2+^	117.35 ± 0.69
Cu^2+^	65.31 ± 1.61
Co^2+^	135.56 ± 3.42
Ni^2+^	100.94 ± 3.04
Ba^2+^	112.36 ± 2.49
Fe^2+^	99.42 ± 2.98

### Kinetic parameters of Man1E

LBG, konjac powder, guar gum, xylan, soluble starch and CMC-Na are used as substrates for evaluating the activity of Man1E. No activity was detected for xylan, soluble starch and CMC-Na (data not shown). Man1E exhibited high activity on LBG, Konjac powder and guar gum, and the catalytic kinetic parameters were determined by Lineweaver–Burk diagram. Man1E showed the highest affinity for LBG, followed successively by Konjac powder and guar gum. The K_m_ values for LBG, Konjac powder and guar gum were 3.09 ± 0.16, 6.07 ± 0.30 and 11.53 ± 0.45 mg/mL, respectively (Additional file 2: Figure S4). The V_max_ of Man1E was 909.10 ± 3.85, 666.67 ± 2.30, and 312.50 ± 4.11 μmol/(mL min) toward LBG, Konjac powder and guar gum, respectively (Additional file 2: Figure S4). The K_m_ value of Man1E for LBG is similar to the *β*-mannanase from *Enterobacter* sp. strain N18, but greater than *β*-mannanase from *K. pneumoniae* SS11 (Table 1). The V_max_ of Man1E is equivalent to *β*-mannanase from *K. pneumoniae* SS11, but lower than *β*-mannanase from *E. asburiae* SD26 (Table 1). Table 3 showed a summary of the kinetic parameters of Man1E.

**Table 3:** The kinetic parameters of Man1E

Substrate	Specific activity (U/mg)	K_m_ (mg/mL)	V_max_ [μmol/(min mL)]
LBG	3028.25 ± 21.33	3.09 ± 0.16	909.10 ± 3.85
Konjac powder	2891.02 ± 32.14	6.07 ± 0.30	666.67 ± 2.30
Guar gum	2198.17 ± 24.68	11.53 ± 0.45	312.50 ± 4.11

### Analysis on hydrolysis products from LBG and KGM

**Fig. 9:**
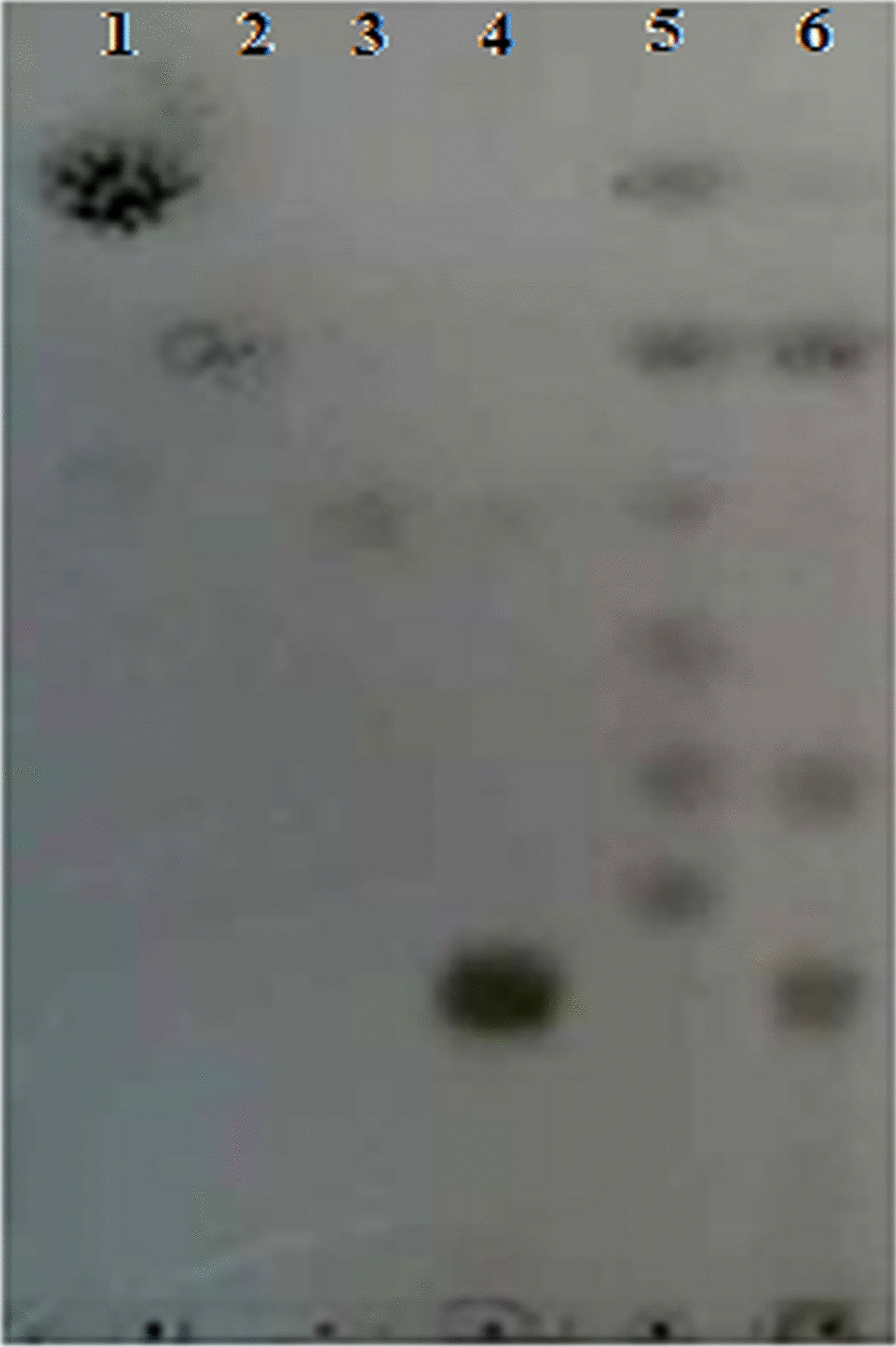
TLC analysis of the products of LBG and KGM hydrolyzed by the purified Man1E. 1, mannose. 2, mannobiose. 3, mannotriose. 4, mannoheptose. 5, products of LBG. 6, products of LBG

TLC analysis of the hydrolysates showed that the purified Man1E could degrade LBG and produced mannanoligosaccharides, which proved Man1E to be an endo-β-mannanase. However, there were obvious differences in the enzymatic degradation products between LBG and KGM. Degradation products of LBG contained mannose to mannohexaose and its components were uniform, while degradation products of KGM was mainly composed of mannobiose, mannopentaose and mannoheptose, and also contained a small amount of mannose and mannotriose (Fig. 9). The hydrolysis of LBG by Man1E was similar to those of β-mannanases from *Bacillus licheniformis* and *B. subtilis* [74].

## Conclusions

To summarise, we have cloned, expressed, and characterized a highly active Man1E from *E. aerogenes*. The amino acid sequence and the molecular mass of Man1E demonstrated significant differences by comparison with all reported β-mannanases in literature, which include several β-mannanases of the Klebsiella-Enterobacter group strains. Unlike previous reports that β-mannanases belong to four families: CH5, CH26, CH133 and CH134, Man1E belonged to CH1 family. Three dimensional structures of the catalytic module and CBM of Man1E were established using homology modeling method, respectively. Important amino acid residues located in the catalytic module and CBM were analyzed by superposition of Man1E and other β-mannanases and molecular docking. The purified recombinant protein showed higher β-mannanase activity. The general properties of the purified enzyme were carefully studied. The enzyme displayed an optimal activity at pH 6.5 and 55 °C. The activity was stable over a broad pH range from acidic to alkaline (3.5–8.5) and below 60 °C, especially below 55 °C. In addition, different metal ions exhibited varied effects on the mannanase activity, Co^2+^ and Mn^2+^ showed better activation. The purified enzyme had a lower K_m_ value to locust bean gum galactomannan, indicating a higher affinity toward locust bean gum galactomannan. The enzyme was an endo-β-mannanase, which displayed excellent catalytic effect on the hydrolysis of locust bean gum galactomannan and Konjac powder glucomannan for the production of oligosaccharides. In future work, it is expected to clarify amino acid sites that play key roles in catalysis and substrate binding, so that Man1E is directionally performed molecular modification to improve its catalytic efficiency and substrate specificity.

## Methods

### Strains, plasmids, and media

*Enterobacter aerogenes* B19 used in this work is now preserved in our laboratory. *Escherichia coli* BL21(DE3) and pET28a(+) acted as the host strain and expression vector, respectively. *Escherichia coli* DH5α was cultured for the amplification of plasmids. *Escherichia* strains were cultivated in Luria–Bertani (LB) medium at 37 °C and 200 r/min in shake flask. Luria–Bertani (LB) medium was composed of 1% NaCl, 0.5% yeast extract, 1% tryptone. LB solid medium contained additional 2% (w/v) agar. 50 µg/L kanamycin was added for screening and culturing the transformants.

### Cloning and sequence analysis of β- Mannanase gene

To obtain the gene encoding Man1E (*Man1E*) from the genomic DNA of *E. aerogenes* B19. 16S rDNA sequence of *E. aerogenes* B19 was blasted in NCBI, and a higher homolog strain (*E. cloacae* P101) was obtained. A β-mannanase gene (GenBank accession no. AHE72721.1) from *E. cloacae* P101 was searched out. Two oligonucleotide primers were designed on the basis of the β-mannanase gene sequence, the upstream primer (Man-F) was 5′-AGCATATGGCTTTCCCGATTCGA-3′ and the downstream primer (Man-R) was 5′-TTCTCGAGCTACTCTGCAGTGACTTCA-3′. The target DNA fragment was amplified by PCR using Man-F and Man-R. The PCR amplification was performed as follows: the first step was to denature the initial template DNA for 5 min at 98 °C, the second step consisted of 30 cycles, each with 10 s at 98 °C, 30 s at 54 °C, 140 s at 68 °C, and the final step was to extend the reaction for 10 min at 72 °C. The objective gene and plasmid pMD19-T (Takara, China) were linked by T4 ligase and the resultant recombinant plasmids were transformed into *E. coli* DH5α. Positive clones were obtained by anti-antibiotic screening, the recombinant pMD19-T –*Man1E* was extracted by a plasmid extraction kit (Axygen, China) and further identified by electrophoresis and sequencing. The analysis for the nucleotide sequence of *Man1E* was conducted using the NCBI ORF Finder tool (https://www.ncbi.nlm.nih.gov/orffinder/). The deduction of the amino acid sequence of Man1E was carried out by EMBOSS Transeq (http://www.ebi.ac.uk/Tools/st/emboss_transeq/), and the molecular weight and isoelectric point of Man1E were predicted using ProtParam (http://web.expasy.org/protparam/). Signal peptide was analyzed using SignalP 4.0 (http://www.cbs.dtu.dk/services/SignalP/). The amino acid sequences of Man1E and other β-mannanases were aligned using Clustal Omega tool (https://www.ebi.ac.uk/Tools/msa/clustalo/).

### Structure prediction and molecular docking analysis

The secondary structure of Man1E was predicted using PSIPRED program (http://bioinf.cs.ucl.ac.uk/psipred/). The tertiary structure of Man1E and Man1ECBM were homology-modeled by the SWISS-MODEL Server (http://swissmodel.expasy.org/) with the crystal structure of *R. miehei* Man5A (4lyp.1.A) (PDB code 4lyp.1.A, 21% identity) as a temple and with the crystal structure of *P. anserina* beta-(1,4)-mannanase CBM35 (PDB code 3zm8.1.A,) as a temple, respectively. The established models were assessed with SAVES v5.0 tool (https://servicesn.mbi.ucla.edu/SAVES/) and Molprobity program (http://molprobity.biochem.duke.edu/index.php).

Mannotriose was used as a substrate molecule (ligand1) and docked into the Man1E catalytic module (receptor1) in the structure model using an AutoDock Tools 4.2.5 (http://autodock.scripps.edu) to detect the interactions between the ligand1 and the receptor1 and the key residues binding to the ligand. Mannopentaose (ligand2) was docked into the Man1ECBM (receptor2) using an AutoDock4 Tools 4.2.5 to investigate the interactions between the ligand2 and the receptor2 and the key residues involved in ligand binding. Molecular visualization and graph drawing were conducted using PyMOL software.

### Expression of *Man1E* in *Escherichia coli* BL21(DE3)

The recombinant pMD19-T –*Man1E* plasmids from the correctly sequenced positive clones and expression vectors pET-28a(+) were digested with NdeI and XhoI, and then linked by DNA ligase to construct the recombinant expression pET-28a(+) –*Man1E* plasmids. The pET-28a(+) –*Man1E* plasmids were transformed into competent cells of *E. coli* BL21(DE3) to express *Man1E*. Positive transformants were screened on LB plate containing 50 μg/mL Kanamycin. At the same time, the pET-28a(+) –*Man1E* recombinant plasmids were extracted from the positive transformants and then digested by NdeI and XhoI, the *Man1E* fragment was further identified by DNA sequencing. The suitable transformants were inoculated in LB medium and cultured for 24 h at 37 °C and 200 r/min. After culture, the cells and supernatant were collected separately for analyzing the recombinant proteins.

### Purification of recombinant Man1E

The fermentation broth was centrifuged at 11,000×*g* and 4 °C for 30 min, the precipitate were harvested and washed with distilled water for three times. The cells was resuspended in 20 mL of 100 mM phosphate buffer (pH 6.5) and the resuspending was ultrasonically disrupted in an ice bath for 20 min. The cell disruption liquid was centrifuged for 30 min at 11,000×*g* and 4 °C. The resultant supernatant was collected and passed through a nickel-chelate column (1 × 5 cm). After equilibration with 100 mM phosphate buffer (pH 6.5), a linear imidazole gradient of 20–250 mM was used to elute the column, Active components were collected and merged, then the solution was dialyzed with 100 mM phosphate buffer (pH 6.5), and stored at 4 °C. The purified protein was analyzed through SDS-PAGE and enzyme activity determination.

### Enzyme activity and protein analysis

The activity of β mannanase was determined by using 3,5-dinitrosalicylic acid (DNS) reagent to measure the amount of reducing sugar released from the substrate. The standard reaction solution consisted of 0.1 mL enzyme liquid with proper dilution and 0.9 mL 0.5% (w/v) substrate solution, which was prepared by dissolving locust bean gum into 100 mM phosphate buffer (pH 6.5). The mixture was incubated at 50 °C for 10 min, then the reaction solution was treated with 1.0 mL DNS reagent to terminate the reaction and boiled for 10 min. The reducing sugar content was determined by measuring its absorbance at 540 nm, taking mannose (sigma) as the standard control to make the standard curve, the relationship formula between the absorbance and the mannose content was obtained. One unit (U) of β-mannanase activity was set to the required enzyme amount that released 1 μmol of reducing sugar (calculated by d-mannose) per minute. Protein content was determined according to Bradford’s method with bovine serum albumin as the standard [75]. The homogeneity and molecular weight of Man1E was investigated by 10% SDS-PAGE as clarified by Laemmli [76]. Protein bands on SDS-PAGE gel were stained with Coomassie brilliant blue R-250. The low molecular weight calibration kit (TaKaRa, China) was used as the molecular weight standard, which included phosphorylase b (97.4 kDa), albumin (66.2 kDa), ovalbumin (43 kDa), carbonic anhydrase (31 kDa), trypsin inhibitor (20.1 kDa), and α-lactalbumin (14.4 kDa).

### Effects of pH, temperature, and metal ions on β-mannanase activity

The optimal catalytic temperature of Man1E was analyzed in a range of 30 and 70 °C in phosphate buffer (pH 6.5). The thermostability of the purified Man1E was investigated by determining the residual activity after the enzyme was preincubated in phosphate buffer (pH 6.5) at different temperatures ranging from 50 °C to 65 °C for 0, 5, 10, 15, 30, 45 and 60 min, respectively. The optimal pH for β-mannanase activity was examined by preparing enzyme solution with different pH buffers which included disodium hydrogen phosphate–citric acid buffer (pH 3.0–6.0), phosphate buffer (pH6.0–8.0), and Tris–HCl buffer (pH 8.0–9.0), the temperature and the substrate used were 50 °C and 0.5% (w/v) locust bean gum. To determine the pH stability, the enzyme was preincubated for 60 min at 40 °C in different pH buffers (pH 3.0–9.0), and the residual activity was measured under the standard conditions. To obtain the effects of metal ions on the purified Man1E, the enzyme was preincubated for 5 min at 40 °C in phosphate buffer (pH 6.5) containing 1 mM single metal ion which contained Na^+^, K^+^, Mn^2+^, Mg^2+^, Zn^2+^, Ca^2+^, Cu^2+^, Ni^2+^, Co^2+^, Ba^2+^, Fe^2+^, separately and then enzyme activity was determined at 50 °C and pH 6.5. The enzyme reaction without any metal ions was used as the control group.

### Kinetic parameters of Man1E

The kinetic parameters were measured using locust bean gum (LBG), guar gum, and KGM as the substrates, respectively. The Michaelis–Menten constant (K_m_) and rate of reaction (V_max_) were calculated from the Lineweaver–Burk plot.

### Analysis of enzymatic hydrolysis products

0.5% (w/V) LBG, KGM were mixed with proper dilution Man1E (final concentration 1.5 U/mL), and the total system volume was 1 mL. The mixture was incubated at pH 6.5 and 55 °C for 8 h, then boiled for 5 min to stop the reaction and the hydrolysates were obtained. The hydrolysates were centrifuged at 12,000×*g* for 10 min. Supernatants were analyzed by using thin layer chromatography (TLC). At room temperature, each sample (0.5 μL) was loaded on the thin layer plate silica gel G). After the plate was dried, it was put into the developing agent composed of n-butanol, acetic acid and water (volume ratio 2:1:1). After chromatography, the monosaccharides and oligosaccharides were determined by aniline- diphenylamine-phosphoric acid- acetone reagent. Mannose, mannobiose, mannotriose and mannoheptose were used as references. The reaction system containing the inactivated Man1E was used as the control.

## Supplementary information

**Additional file 1: Figure S1.** The 3D structure of the catalytic active domains (CD) and family 35 carbohydrate binding modules (CBM) of β-1, 4-mannanases. **a** A GH5 family β-1, 4-mannanase from *Podospora Anserina* (PDB code, 3ZM8). **b** The CBM (PDB code, 2WZ8) of β-1, 4-mannanase from *Podospora Anserina* (PDB code, 3ZM8). **c** A GH26 family β-1,4-mannanase from *Cellulomonas fimi* (PDB code, 2BVT). **d** The CBM of a GH5 family β-1, 4-mannanase from the *Cellvibrio japonicus* (PDB code, 2BGP). **e** The resultant conformation of Man1E docking TRS.

**Additional file 2: Figure S2.** Multiple sequence alignment of Man1E with other mannan-degrading enzymes. Unpublished β-1, 4-mannanases from *Klebsiella aerogenes* (NCBI accession No., WP_108418545.1), *Enterobacter ludwigii* (NCBI accession No., WP_086532142.1), *Escherichia coli* (NCBI accession No., WP_160515149.1), *Solanum lycopersicum* (NCBI accession No., Q6YM50.1), *Oryza sativa Japonica* Group (NCBI accession No., Q0JKM9.2) and *Arabidopsis thaliana* (NCBI accession No., Q9FZ29.1). Published GH5 family β-1, 4-mannanases from *Cellvibrio mixtus* (PDB code, 1UUQ-A), *Rhizomucor miehei* (PDB code, 4LYP-A), *Solanum lycopersicum* (PDB code, 1RH9-A), *Podospora Anserina* (PDB code, 3ZIZ-A) and *Trichoderma Reesei* (PDB code, 1QNR-A). **Figure S3.** The conserved domains of Man1E predicted with NCBI CDD Tool. **Figure S4.** Lineweaver–Burk double reciprocal plots of β-mannanase from *Enterobacter aerogenes* B19 against LBG (**a**), konjac powder (**b**), guar gum (**c**). The K_m_ and V_max_ were calculated from the Lineweaver-Burke plot.

## Data Availability

All data generated or analyzed during this study are included in this published article and its additional file.

## Abbreviations

Man1E: β-mannanase from *Enterobacter aerogenes* B19
*Man1E*: β-mannanase gene from *Enterobacter aerogenes* B19
CDD: Conserved domain database
Man1ECBM: CBM6-CBM35-CBM36_like domain of Man1E
Mw: Molecular weight
GH: Glycosyl hydrolase
LBG: Locust bean gum
KGM: Konjac glucomannan
IPTG: Isopropylthiogalactoside
