# Supplementary material for: Expression, homology modeling and enzymatic characterization of a new β-mannanase belonging to glycoside hydrolase family 1 from Enterobacter aerogenes B19
Source: Microb Cell Fact. 2020 Jul 14;19:142. doi: 10.1186/s12934-020-01399-w (PMC7362650; doi:10.1186/s12934-020-01399-w)
Supplement: Supplementary file 2 — Additional file 2: Figure S2. Multiple sequence alignment of Man1E with other mannan-degrading enzymes. Unpublished β-1, 4-mannanases from Klebsiella aerogenes (NCBI accession No., WP_108418545.1), Enterobacter ludwigii (NCBI accession No., WP_086532142.1), Escherichia coli (NCBI accession No., WP_160515149.1), Solanum lycopersicum (NCBI accession No., Q6YM50.1), Oryza sativa Japonica Group (NCBI accession No., Q0JKM9.2) and Arabidopsis thaliana (NCBI accession No., Q9FZ29.1). Published GH5 family β-1, 4-mannanases from Cellvibrio mixtus (PDB code, 1UUQ-A), Rhizomucor miehei (PDB code, 4LYP-A), Solanum lycopersicum (PDB code, 1RH9-A), Podospora Anserina (PDB code, 3ZIZ-A) and Trichoderma Reesei (PDB code, 1QNR-A). Figure S3. The conserved domains of Man1E predicted with NCBI CDD Tool. Figure S4. Lineweaver–Burk double reciprocal plots of β-mannanase from Enterobacter aerogenes B19 against LBG (a), konjac powder (b), guar gum (c). The Km and Vmax were calculated from the Lineweaver-Burke plot. [file 12934_2020_1399_MOESM2_ESM.docx]

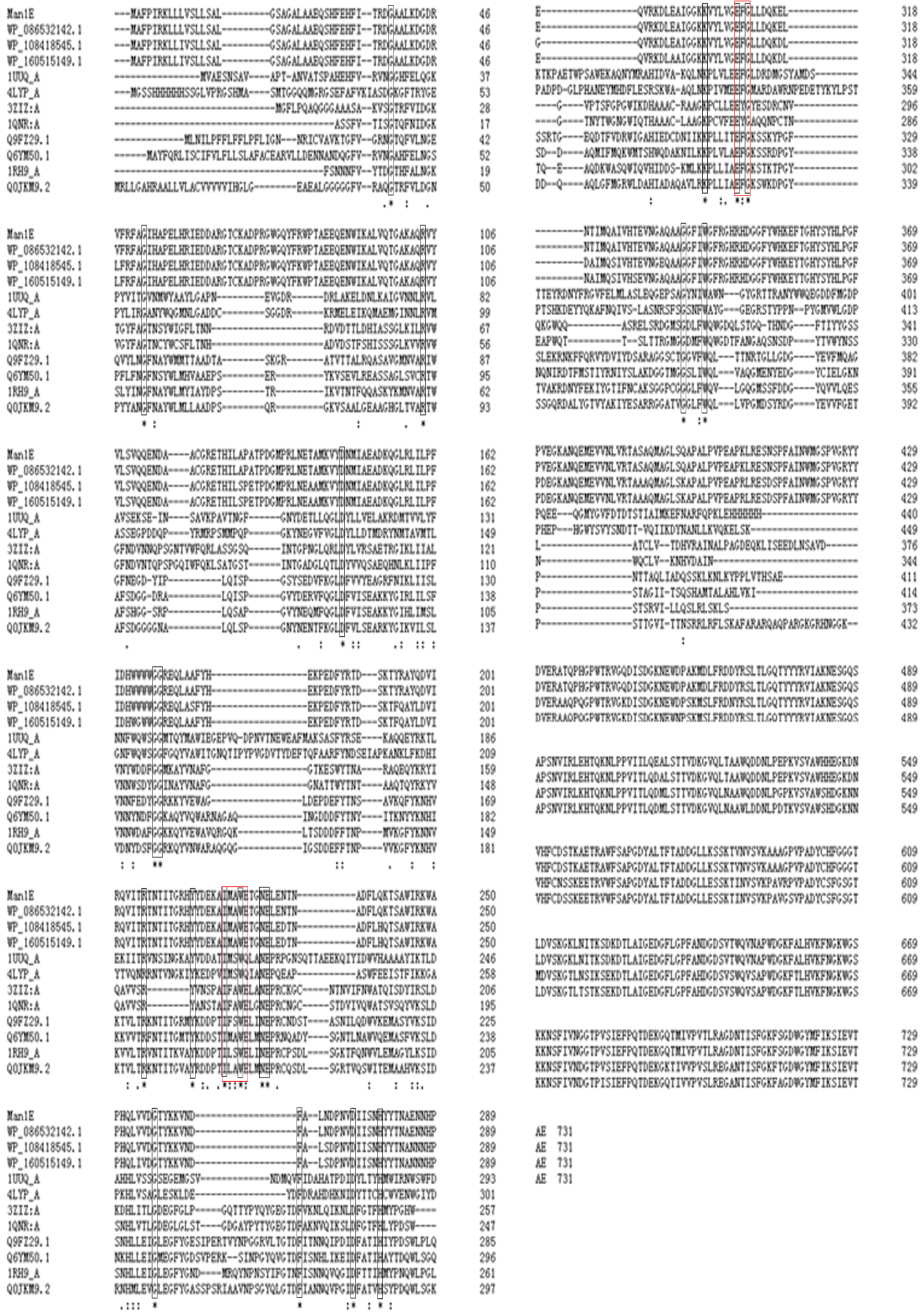


**Figure S2** Multiple sequence alignment of Man1E with other mannan-degrading enzymes. Unpublished β-1, 4-mannanases from *Klebsiella aerogenes* (NCBI accession No., WP_108418545.1), *Enterobacter ludwigii* (NCBI accession No., WP_086532142.1), *Escherichia coli* (NCBI accession No., WP_160515149.1), *Solanum lycopersicum* (NCBI accession No., Q6YM50.1), *Oryza sativa Japonica* Group (NCBI accession No., Q0JKM9.2) and *Arabidopsis thaliana* (NCBI accession No., Q9FZ29.1). Published GH5 family β-1, 4-mannanases from *Cellvibrio mixtus* (PDB code, 1UUQ-A), *Rhizomucor miehei* (PDB code, 4LYP-A), *Solanum lycopersicum* (PDB code, 1RH9-A), *Podospora Anserina* (PDB code, 3ZIZ-A) and *Trichoderma Reesei* (PDB code, 1QNR-A).


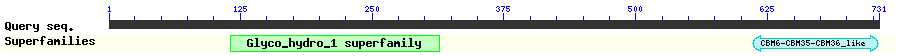


**Figure S3** The conserved domains of Man1E predicted with NCBI CDD Tool.


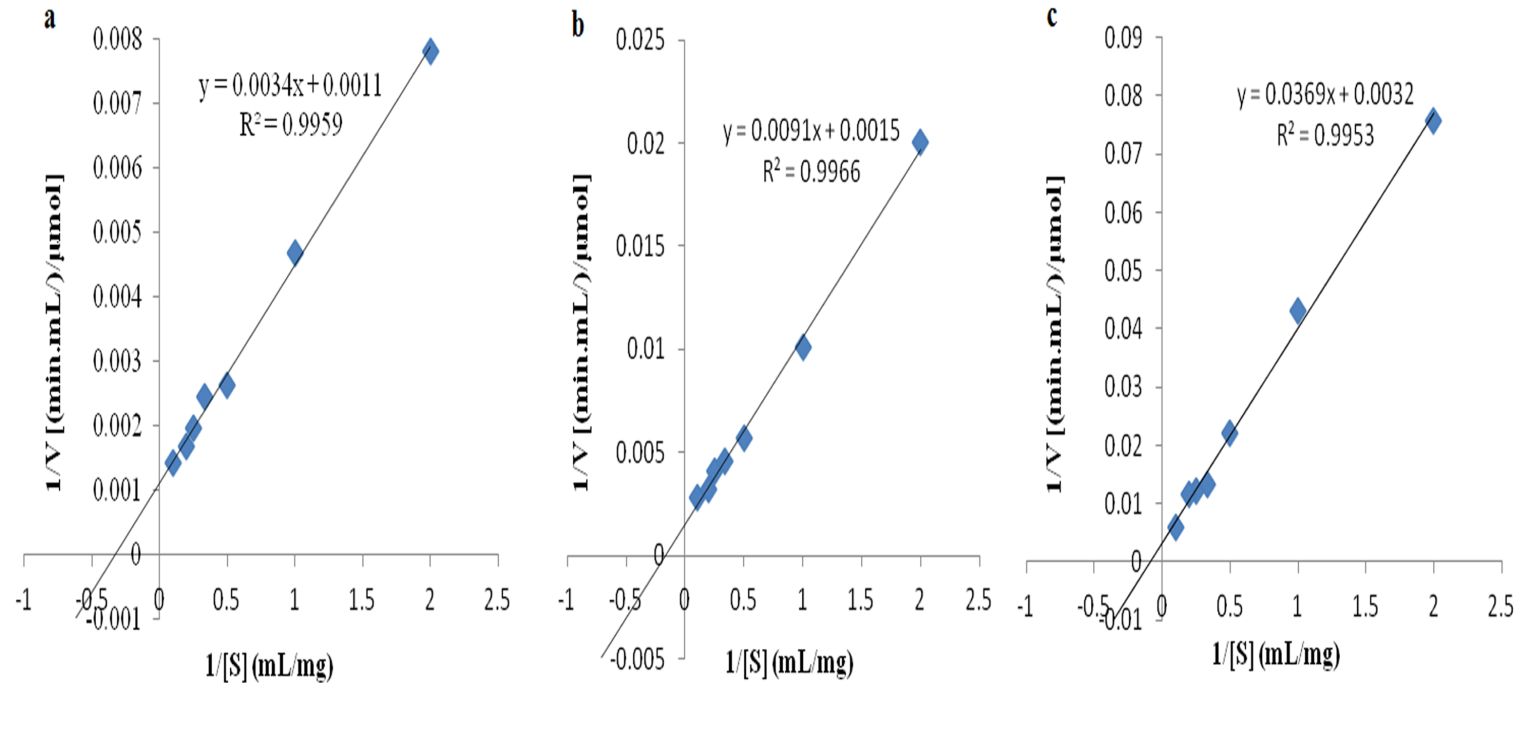
 **Figure S4** Lineweaver-Burk double reciprocal plots of β-mannanase from *Enterobacter aerogenes* B19 against LBG (**a**), konjac powder (**b**), guar gum (**c**). The K_m_ and V_max_ were calculated from the Lineweaver-Burke plot.
